# Development of a machine learning algorithm model to predict intraoperative hypotension in elderly patients undergoing thoracic and abdominal surgeries

**DOI:** 10.1515/med-2026-1381

**Published:** 2026-03-16

**Authors:** Yifan An, Pengfei Liu, Lei Liu, Xiaoyun Hu, Hui Qiao, Weixuan Sheng

**Affiliations:** Department of Anesthesiology, Beijing Shijitan Hospital, Capital Medical University, Beijing, China; Department of Science and Technology, Beijing Shijitan Hospital, Capital Medical University, Beijing, China

**Keywords:** intraoperative hypotension, machine learning, confusion matrix, SHapley Additive exPlanations, the elderly

## Abstract

**Objectives:**

To develop and validate machine learning (ML) models for identifying key predictors and estimating the risk of intraoperative hypotension (IOH) in elderly patients undergoing general anesthesia.

**Methods:**

This secondary analysis included 1,720 elderly surgical patients from a randomized controlled trial. Data were split chronologically into training sets. Feature selection was performed using univariate analysis and the Boruta algorithm. Eight ML models – logistic regression, Bayesian model, K-nearest neighbor, support vector machine, neural network, classification and regression tree, extreme gradient boosting, and random forest – were developed with cross-validation, hyperparameter tuning, and random oversampling. Model performance was evaluated using ROC, PRC, calibration, and decision curve analyses, and interpretability was enhanced using SHapley Additive exPlanations (SHAP).

**Results:**

Key predictors included anesthesia protocol, Charlson comorbidity index, preoperative sodium, creatinine, BUN/creatinine ratio, intraoperative drug use (e.g., sevoflurane, lidocaine, morphine), preoperative MAP and MHR, surgical and anesthesia duration, and surgical site. The random forest model achieved the best performance (accuracy=0.9917; MCC=0.9832; AUC-ROC=0.9998; AUC-PRC=0.9998).

**Conclusions:**

A robust ML-based model was established to accurately predict IOH in elderly patients. These findings may support individualized anesthesia management and targeted preventive strategies to reduce IOH incidence.

## Introduction

Arterial blood pressure is one of the most critical hemodynamic parameters during the perioperative period [1], 2]. Previous observational studies have established associations between intraoperative hypotension (IOH) and adverse outcomes in both noncardiac and cardiac surgeries [3], [4], [5]. Hypotensive events have been linked to myocardial injury [6] and mortality [3], 7] in noncardiac surgery. In cardiac surgeries, a 10 % reduction in mean arterial pressure (MAP) from baseline during cardiopulmonary bypass correlates with increased stroke risk, and each additional minute of intraoperative hypotension elevates the postoperative stroke risk by 1.013-fold [8]. Wijnberge et al. reported that 10 min of MAP <80 mmHg, shorter durations of MAP <70 mmHg, or any exposure to MAP <55 mmHg were associated with end-organ damage in non-cardiac surgeries [9]. IOH is particularly prevalent in elderly populations. Older adults often present with comorbidities, such as arteriosclerosis, coronary artery disease, and hypertension, along with declining cardiopulmonary and autonomic functions [10], 11]. These factors, combined with the hemodynamic effects of anesthesia and surgical procedures, increase the susceptibility to IOH [12]. Therefore, it is imperative to develop precise predictive models for the risk of IOH in elderly patients.

Effective prediction of IOH in elderly patients remains a global research priority [12]. Clinical anesthesia generates vast and complex datasets that traditional methods struggle to fully utilize [13]. Previous studies have predominantly employed logistic regression, which cannot process visual data (e.g., vital sign curves) or time-series data effectively [14], thus limiting real-time IOH prediction [13]. However, machine learning (ML) excels at uncovering latent relationships in multidimensional data and enhancing model accuracy and calibration. This study used multimodal data and deep learning to construct and validate an IOH prediction model for elderly patients undergoing thoracic/abdominal surgery, aiming to identify risk factors and inform clinical prevention strategies.

## Materials and methods

### Study population and data collection

Data were derived from the randomized trial *“Delirium in Older Patients after Combined Epidural-General Anesthesia or General Anesthesia for Major Surgery”* [original trial details]. This secondary analysis utilized a dataset approved by the Peking University Institutional Review Board (No. 00001052-11048) and the ethics committees of the five participating centers. The trial was registered with the Chinese Clinical Trial Registry (www.chictr.org.cn; identifier: ChiCTR-TRC-90000543) and ClinicalTrials.gov (NCT01661907).

The original trial enrolled patients aged 60–90 years undergoing elective non-cardiac thoracic/abdominal surgeries (≥2 h duration) requiring postoperative patient-controlled analgesia. The exclusion criteria included severe neurological disorders, acute myocardial infarction/stroke within 3 months, severe cardiac/hepatic/renal dysfunction, or contraindications to epidural anesthesia.

### Definition of intraoperative hypotension

IOH was defined as: absolute systolic blood pressure (SBP) <80 mmHg [15], or relative SBP reduction >20 % from preoperative baseline, sustained ≥1 min [16].

### Variables

#### Predictors

55 potential features included:

Demographics: age, sex, education years, BMI, ASA class (American Society of Anesthesiologists physical status classification).

Anesthesia protocol: general anesthesia alone with patient-controlled intravenous analgesia vs. general epidural anesthesia combined with patient-controlled epidural analgesia (PCEA).

Cognitive/psychological scores: Mini Mental State Examination, depression/anxiety scores.

Comorbidity indices: Charlson comorbidity index and preoperative laboratory results (hematocrit, albumin, glucose, sodium, potassium, creatinine [CREA], blood urea nitrogen [BUN], and BUN/CREA ratio).

Comorbidities included stroke, transient ischemic attack, chronic obstructive pulmonary disease, chronic bronchitis, asthma, coronary artery disease, hypertension, arrhythmia, diabetes, thyroid disorders, hepatic/renal insufficiency, and hyperlipidemia.

The intraoperative data included nitrous oxide, sevoflurane, midazolam, atropine, antiemetics, nonsteroidal anti-inflammatory drugs, lidocaine, ropivacaine, total morphine equivalents, fluid administration (crystalloids, colloids, red blood cells, and plasma), blood loss, and urine output.

Hemodynamics: preoperative mean arterial pressure (MAP), preoperative mean heart rate (MHR). At 9 time points on the day before surgery, including: 6:00 (upon waking), 8:00 (during breakfast), 10:00 (daytime activity), 12:00 (lunch), 14:00 (afternoon), 16:00 (late afternoon), 18:00 (dinner), 20:00 (after dinner), and 22:00 (before bedtime), the patient’s systolic blood pressure, diastolic blood pressure, and heart rate were measured. Prior to each measurement, the patient rested in a seated position for 5 min. At each time point, two consecutive measurements were taken. The mean arterial pressure and heart rate for each of the 9 time points were then calculated, and their respective averages were determined to obtain the preoperative mean blood pressure and mean heart rate [17].

Surgical details included duration (surgery/anesthesia), surgical site (abdominal/thoracic), and laparoscopic approach.

#### Outcome

Binary variable: occurrence of IOH (yes/no).

### Statistical analysis and sample size

The analyses were performed using R (v4.2.2) and RStudio (v2023.06.0+421). Continuous variables were tested for normality using the Shapiro-Wilk test. Normally distributed variables are reported as mean ± SD; non-normal variables as median (interquartile range). Categorical data were presented as frequencies (%).

The dataset was split into training (between January 2015 and December 2016, n=1,119) and testing (January 2017 and June 2018, n=601) sets based on time. Feature selection in the training set was performed using univariate analysis and the Boruta algorithm. Eight ML models – logistic regression (LR), Bayes model, K-nearest neighbor (KNN), support vector machine (SVM), neural network (NNET), the classification and regression tree (CART), extreme gradient boosting (XGBoost), and random forest (RF) – were trained and optimized using repeated cross validation, hyperparameter tuning, and random oversampling (mlr3verse package). The optimal model was interpreted using SHapley Additive exPlanations (SHAP) for feature importance ranking, partial dependence plots, and force plots. Performance was validated on the testing set using confusion matrix metrics, receiver operating characteristics (ROC)/precision recall (PRC) curves, calibration curves, and decision curve analysis.

Based on the rule of 10 events per predictor – which requires at least 10 positive events (intraoperative hypotension, IOH cases) for each predictor variable included in the final model – this study planned to incorporate 55 predictors. Consequently, a minimum of 550 IOH cases was required. Given the reported IOH incidence rate of 25–60 % [18], 19], the calculated sample size for the modeling cohort ranged from 916 to 2,200 patients. This study enrolled 1,119 elderly surgical patients as the training set, ensuring the requirement for model development, and subsequently included 601 patients as the validation set for model verification.

### Ethical statement

This secondary analysis utilized a dataset approved by the Peking University Institutional Review Board (No. 00001052-11048) and the ethics committees of the five participating centers. The trial was registered with the Chinese Clinical Trial Registry (www.chictr.org.cn; identifier: ChiCTR-TRC-90000543) and ClinicalTrials.gov (NCT01661907).

## Results

### Statistical flowchart and baseline characteristics

**Figure 1:**
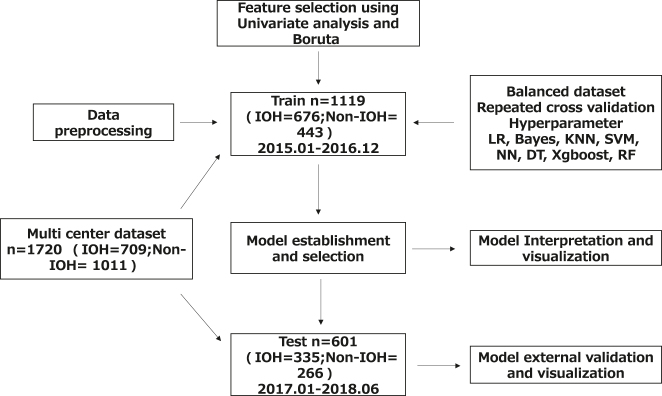
The statistical flowchart of clinical data. IOH, intraoperative hypotension; LR, logistic regression; Bayes, Bayes model; KNN, K-nearest neighbors; SVM, support vector machine; NN, neural network; DT, decision tree; Xgboost, extreme gradient boosting; RF, random forest.

The dataset included clinical data from 1,720 patients. The workflow for data inclusion, model development, selection, interpretation, and external validation is shown in Figure 1. The baseline characteristics of the training and testing sets are summarized in Table 1. The optimal model was selected and validated after repeated k-fold cross-validation and hyperparameter optimization. The incidence of IOH was 60.41 % (676/1,119) and 55.74 % (335/601) in the training and testing sets, respectively. Comparisons of preoperative, intraoperative, and postoperative variables between the IOH and non-IOH groups are shown in Table 1.

**Table 1:** Univariate analysis of clinical data (n=1720).

Data set		Train (n=1,119)		Test (n=601)	
Factors		Non-IOH (n=676)	IOH (n=443)	Non-IOH (n=335)	IOH (n=266)
Age, year		69.00 [64.00, 75.00]	68.00 [64.00, 74.00]	69.00 [65.00, 74.00]	68.00 [64.00, 74.00]
Years of education		9.00 [6.00, 13.00]	9.00 [6.00, 12.00]	9.00 [6.00, 12.00]	9.00 [6.00, 12.75]
BMI		23.88 [21.71, 25.71]	23.24 [20.98, 25.77]^a^	23.81 [22.00, 26.28]	23.21 [21.11, 25.50]^a^
MMSE score		29.00 [27.00, 30.00]	29.00 [27.00, 30.00]	29.00 [28.00, 30.00]	29.00 [28.00, 30.00]
Anxiety score		0.00 [0.00, 2.00]	0.00 [0.00, 1.50]^a^	0.00 [0.00, 2.00]	0.00 [0.00, 2.00]
Depression score		1.00 [0.00, 3.00]	0.00 [0.00, 2.00]^a^	1.00 [0.00, 2.00]	0.00 [0.00, 1.75]^a^
CHARLSON score		78.04 [50.00, 120.00]	121.00 [51.00, 121.00]^a^	67.00 [50.00, 120.00]	121.00 [51.00, 121.00]^a^
Hct		38.35 [34.80, 41.80]	38.70 [34.90, 42.20]	38.90 [35.70, 41.35]	38.30 [35.70, 41.58]
ALB		40.20 [37.30, 43.12]	40.20 [36.70, 42.85]	41.70 [38.40, 43.45]	40.70 [37.70, 43.27]
GLU		5.40 [4.89, 6.22]	5.32 [4.80, 5.96]	5.45 [4.99, 6.14]	5.39 [4.86, 6.24]
Na		142.00 [140.00, 143.30]	141.50 [139.70, 143.00]^a^	141.40 [140.00, 143.00]	142.00 [140.00, 143.60]^a^
K		4.05 [3.80, 4.32]	4.05 [3.77, 4.31]	3.94 [3.68, 4.24]	3.98 [3.67, 4.25]
CREA		85.00 [73.00, 99.00]	87.00 [76.00, 99.00]^a^	88.00 [76.00, 98.00]	86.00 [77.00, 99.75]
BUN		5.77 [4.70, 6.82]	5.58 [4.58, 6.81]	5.59 [4.68, 6.93]	5.30 [4.39, 6.55]^a^
BUN/CREA		16.53 [13.58, 20.62]	16.04 [12.94, 19.14]^a^	15.82 [13.48, 19.23]	15.30 [12.62, 17.87]^a^
N_2_O, %		1.00 [0.00, 2.00]	1.00 [0.00, 2.00]	1.00 [0.00, 2.00]	1.00 [0.00, 2.00]
Sevoflurane, %		1.00 [0.00, 1.00]	0.80 [0.00, 1.00]^a^	0.00 [0.00, 1.00]	0.00 [0.00, 1.00]^a^
Midazolam, mg		2.00 [1.00, 2.00]	1.50 [1.00, 2.00]^a^	1.50 [1.00, 2.00]	1.50 [1.05, 2.00]^a^
Lidocaine, mg		0.00 [0.00, 60.00]	40.00 [0.00, 60.00]^a^	0.00 [0.00, 40.00]	0.00 [0.00, 60.00]^a^
Ropivacaine, mg		0.00 [0.00, 80.00]	50.00 [0.00, 100.00]^a^	0.00 [0.00, 69.00]	40.00 [0.00, 100.00]^a^
Crystal liquid, mL		1850.00 [1,537.50, 2,450.00]	1950.00 [1,600.00, 2,575.00]	1850.00 [1,600.00, 2,600.00]	2,100.00 [1,600.00, 2,700.00]
Colloid liquid, mL		500.00 [500.00, 1,000.00]	500.00 [500.00, 1,000.00]	500.00 [0.00, 500.00]	500.00 [500.00, 1,000.00]^a^
Red blood cells, mL		0.00 [0.00, 0.00]	0.00 [0.00, 0.00]	0.00 [0.00, 0.00]	0.00 [0.00, 0.00]
Plasma, mL		0.00 [0.00, 0.00]	0.00 [0.00, 0.00]	0.00 [0.00, 0.00]	0.00 [0.00, 0.00]^a^
Urine volume, mL		400.00 [137.50, 700.00]	400.00 [200.00, 650.00]	350.00 [100.00, 600.00]	300.00 [100.00, 600.00]
Bleeding volume, mL		150.00 [50.00, 400.00]	100.00 [50.00, 300.00]	100.00 [50.00, 200.00]	100.00 [50.00, 300.00]^a^
Preoperative MAP, mmHg		83.47 [78.96, 88.11]	77.07 [72.76, 81.88]^a^	82.67 [77.41, 87.89]	75.75 [72.74, 80.17]^a^
Preoperative MHR, times/minute		64.69 [59.19, 72.38]	70.11 [63.99, 76.65]^a^	64.24 [59.20, 70.74]	68.91 [62.14, 74.76]^a^
Duration of anesthesia, min		271.44 [212.60, 349.16]	303.08 [238.11, 386.33]^a^	271.44 [212.35, 345.36]	303.60 [238.85, 384.64]^a^
Duration of surgery, min		218.50 [160.00, 290.00]	248.00 [183.50, 322.00]^a^	220.00 [159.00, 285.00]	247.50 [180.00, 329.00]^a^
The total intraoperative dosage of opioids, mg		182.50 [145.00, 255.00]	165.00 [141.67, 223.50]^a^	206.00 [155.00, 260.00]	196.50 [150.00, 263.75]
Gender	Male	429 (63.5)	302 (68.2)	223 (66.6)	169 (63.5)
	Female	247 (36.5)	141 (31.8)	112 (33.4)	97 (36.5)
ASA	ASA-I	49 (7.2)	26 (5.9)^a^	34 (10.1)	14 (5.3)^a^
	ASA-II	559 (82.7)	391 (88.3)	284 (84.8)	238 (89.5)
	ASA-III	68 (10.1)	26 (5.9)	17 (5.1)	14 (5.3)
Group	GEA-PCEA	382 (56.5)	179 (40.4)^a^	193 (57.6)	109 (41.0)^a^
	GA-PCIA	294 (43.5)	264 (59.6)	142 (42.4)	157 (59.0)
Stroke	Yes	31 (4.6)	21 (4.7)	19 (5.7)	14 (5.3)
	No	645 (95.4)	422 (95.3)	316 (94.3)	252 (94.7)
TIA	Yes	15 (2.2)	1 (0.2)^a^	3 (0.9)	4 (1.5)
	No	661 (97.8)	442 (99.8)	332 (99.1)	262 (98.5)
COPD	Yes	10 (1.5)	11 (2.5)	6 (1.8)	5 (1.9)
	No	666 (98.5)	432 (97.5)	329 (98.2)	261 (98.1)
Chronic bronchitis	Yes	10 (1.5)	7 (1.6)	9 (2.7)	6 (2.3)
	No	666 (98.5)	436 (98.4)	326 (97.3)	260 (97.7)
Asthma	Yes	9 (1.3)	7 (1.6)	5 (1.5)	6 (2.3)
	No	667 (98.7)	436 (98.4)	330 (98.5)	260 (97.7)
Smoke	Yes	165 (24.4)	130 (29.3)^a^	61 (18.2)	60 (22.6)
	No	511 (75.6)	313 (70.7)	274 (81.8)	206 (77.4)
Coronary heart disease	Yes	85 (12.6)	33 (7.4)^a^	25 (7.5)	23 (8.6)
	No	591 (87.4)	410 (92.6)	310 (92.5)	243 (91.4)
Hypertension	Yes	297 (43.9)	165 (37.2)^a^	150 (44.8)	99 (37.2)^a^
	No	379 (56.1)	278 (62.8)	185 (55.2)	167 (62.8)
Arrhythmia	Yes	25 (3.7)	16 (3.6)	14 (4.2)	8 (3.0)
	No	651 (96.3)	427 (96.4)	321 (95.8)	258 (97.0)
NYHA	I	485 (71.7)	347 (78.3)^a^	250 (74.6)	217 (81.6)^a^
	II	191 (28.3)	96 (21.7)	85 (25.4)	49 (18.4)
Diabetes	Yes	132 (19.5)	72 (16.3)	65 (19.4)	45 (16.9)
	No	544 (80.5)	371 (83.7)	270 (80.6)	221 (83.1)
Thyroid diseases	Yes	14 (2.1)	9 (2.0)	8 (2.4)	14 (5.3)
	No	662 (97.9)	434 (98.0)	327 (97.6)	252 (94.7)
Abnormal liver function	Yes	5 (0.7)	3 (0.7)	5 (1.5)	2 (0.8)
	No	671 (99.3)	440 (99.3)	330 (98.5)	264 (99.2)
Hyperlipidemia	Yes	22 (3.3)	13 (2.9)	6 (1.8)	5 (1.9)
	No	654 (96.7)	430 (97.1)	329 (98.2)	261 (98.1)
Abnormal renal function	Yes	1 (0.1)	3 (0.7)	2 (0.6)	1 (0.4)
	No	675 (99.9)	440 (99.3)	333 (99.4)	265 (99.6)
Drinking alcohol	Yes	143 (21.2)	105 (23.7)	91 (27.2)	87 (32.7)
	No	533 (78.8)	338 (76.3)	244 (72.8)	179 (67.3)
Atropine	Given	486 (71.9)	344 (77.7)^a^	247 (73.7)	207 (77.8)
	Not given	190 (28.1)	99 (22.3)	88 (26.3)	59 (22.2)
Antiemetic medicine	Given	600 (88.8)	396 (89.4)	294 (87.8)	238 (89.5)
	Not given	76 (11.2)	47 (10.6)	41 (12.2)	28 (10.5)
NSAIDs	Given	582 (86.1)	375 (84.7)	229 (68.4)	163 (61.3)
	Not given	94 (13.9)	68 (15.3)	106 (31.6)	103 (38.7)
Surgical sites	Abdomen	573 (84.8)	291 (65.7)^a^	278 (83.0)	174 (65.4)^a^
	Chest	103 (15.2)	152 (34.3)	57 (17.0)	92 (34.6)
Endoscope	Given	473 (70.0)	339 (76.5)^a^	176 (52.5)	180 (67.7)^a^
	Not given	203 (30.0)	104 (23.5)	159 (47.5)	86 (32.3)

^a^p<0.1 is used as the condition for single-factor screening of independent variables.

In the training set, the IOH group exhibited significantly lower values than the non-IOH group for BMI, anxiety/depression scores, serum sodium, blood urea nitrogen (BUN), BUN/creatinine (CREA) ratio, sevoflurane concentration, midazolam dosage, preoperative MAP, total intraoperative opioid equivalents, ASA-I classification proportion, combined general-epidural anesthesia with PCEA (GEA-PCEA) proportion, transient ischemic attack prevalence, coronary artery disease prevalence, hypertension prevalence, and abdominal surgery proportion (p<0.1). Conversely, the IOH group showed a significantly higher Charlson comorbidity index, CREA levels, lidocaine/ropivacaine dosage, preoperative MHR, anesthesia duration, surgical duration, smoking prevalence, New York Heart Association (NYHA) class I proportion, atropine usage, and laparoscopic surgery proportion (p<0.1).

In the testing set, the IOH group had lower BMI, depression scores, BUN/CREA ratio, sevoflurane concentration, midazolam dosage, preoperative MAP, ASA-I proportion, GEA-PCEA proportion, hypertension prevalence, and abdominal surgery proportion (p<0.1). Conversely, the IOH group showed higher Charlson comorbidity index, serum sodium, CREA levels, lidocaine/ropivacaine dosage, preoperative MHR, anesthesia/surgical duration, smoking prevalence, NYHA class I proportion, and laparoscopic surgery proportion (p<0.1).

### Feature selection

**Figure 2:**
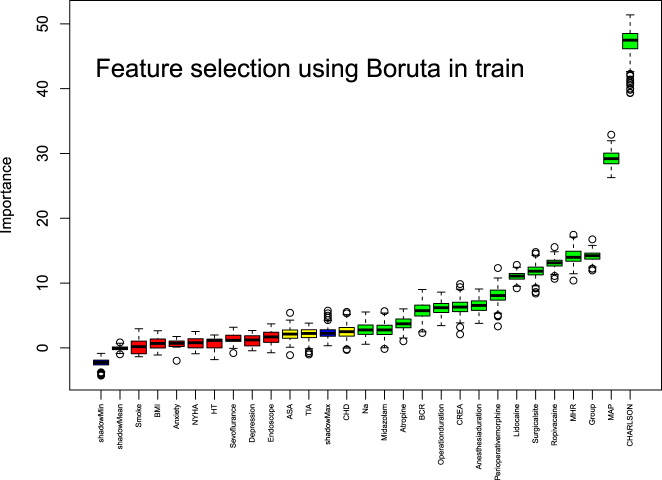
Screening of Boruta characteristic variables. This graph illustrates the importance of feature variables as determined by the Boruta algorithm. The independent variables on the right side of ShadowMax are the feature variables filtered by Boruta. The x-axis displays the individual feature variables, while the y-axis represents their importance scores.

Univariate analysis (p<0.1) and Boruta algorithm identified 16 key predictors: anesthesia protocol, Charlson comorbidity index, preoperative laboratory results (serum Na, CREA, BUN/CREA ratio), intraoperative medications (sevoflurane, midazolam, atropine, lidocaine, ropivacaine, total morphine equivalents), preoperative MAP, preoperative MHR, surgical/anesthesia duration, and surgical site (Figure 2).

### Model internal validation

**Figure 3:**
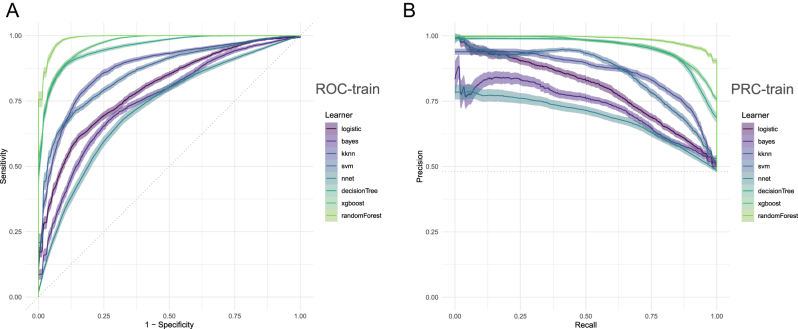
The ROC, and PRC of the clinical data (A) represented ROC of eight models; (B) represented PRC of eight models. The figure shows the process of model selection. Figure 3 shows the performance of eight models in terms of ROC (A) and PRC (B). Among them, RF has the highest AUC-ROC (A) and AUC-PRC (B) values.

After determining these 16 variables, machine learning models were applied to predict intraoperative hypotension (IOH). The area under the receiver operating characteristic curve (AU-ROC) and the area under the precision-recall curve (AUC-PRC) serve as crucial metrics for evaluating prediction models. Among the eight models established, the random forest (RF) model demonstrated the best performance across ROC and PRC metrics shown in Figure 3. Figure 3A and B shows that RF attained the highest AUC-ROC and AUC-PRC values among the eight models. These findings indicated that the random forest model performed exceptionally well in accuracy, overall performance, general discriminative power, and detection of positive results.

### Model external validation

**Figure 4:**
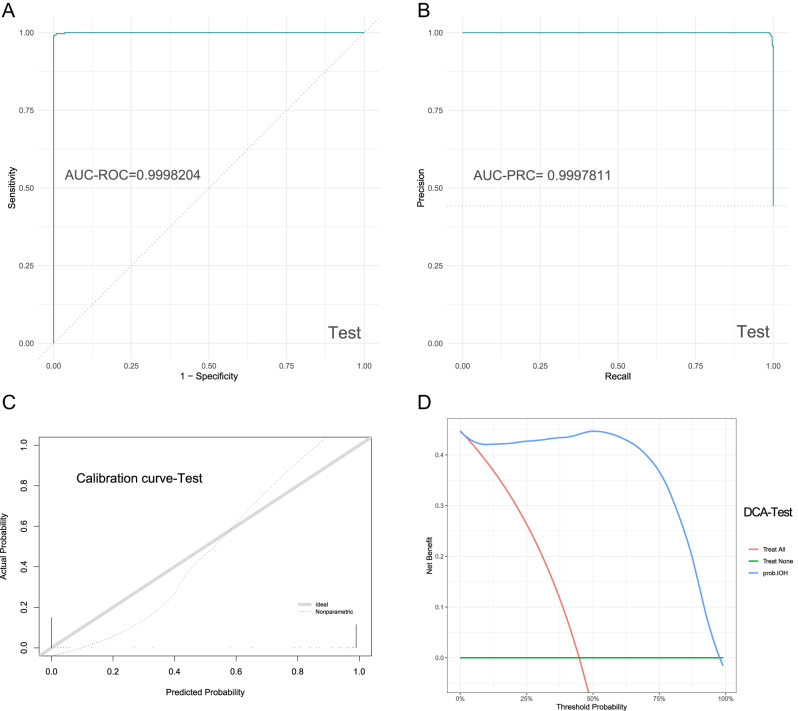
ROC, PRC, calibration curve, and DCA. (A) represented ROC of the test set; (B) represented PRC of the test set; (C) represented calibration curve of the test set; (D) represented DCA of the test set.

External validation was similarly conducted on the machine learning model for intraoperative hypotension prediction. The confusion matrix parameters for the test set were as follows: Accuracy: 0.9916805; MCC: 0.9832086; AUC-ROC: 0.9998204; AUC-PRC: 0.9997811. The ROC curve, PRC curve, calibration curve, and DCA (decision curve analysis) for the test set are presented in Figure 4. These results demonstrate that the random forest model exhibits outstanding performance in accuracy, overall performance, discriminative power, and positive predictive capability.

### Feature importance plots, force plots, and partial dependence plots

**Figure 5:**
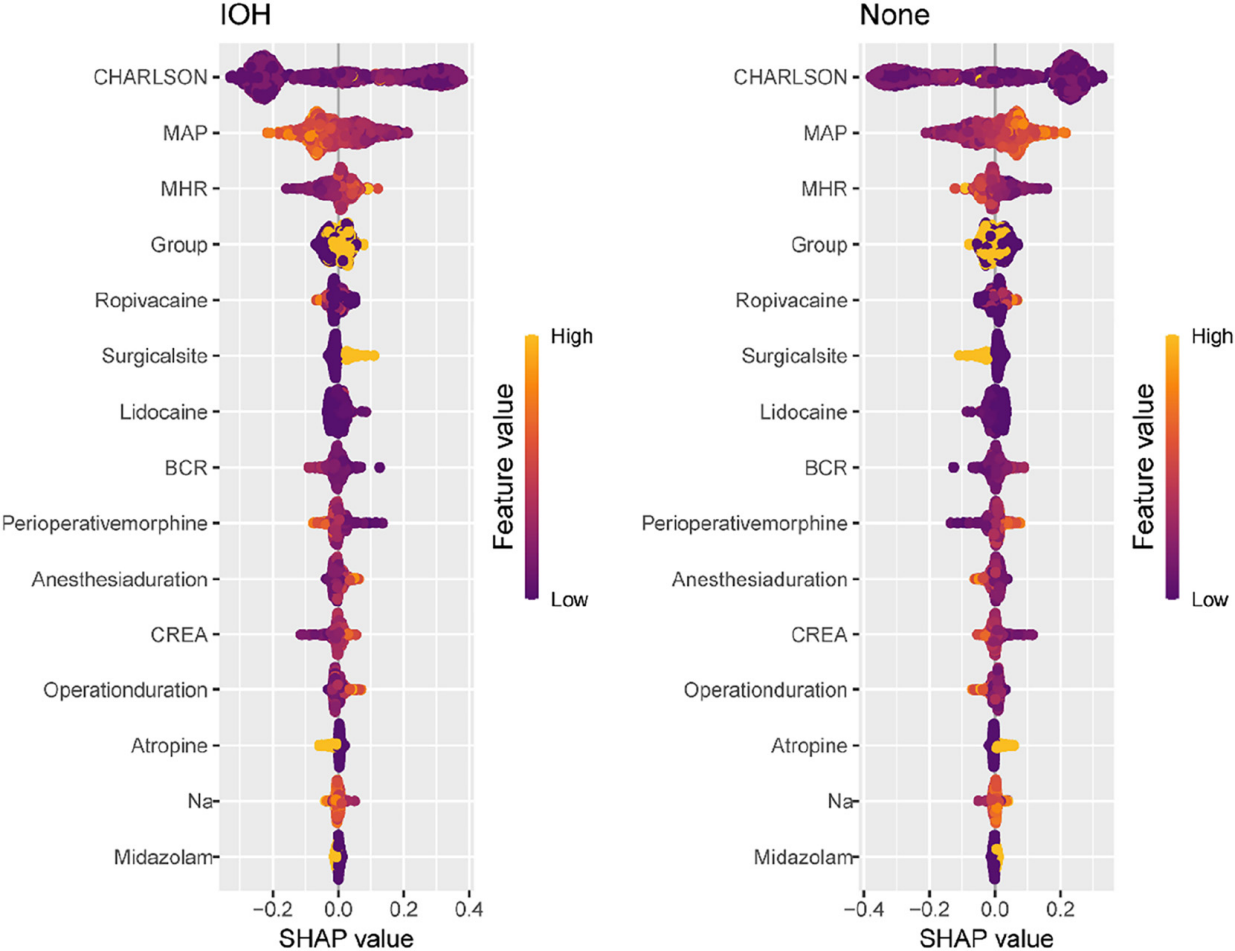
Importance ranking of characteristic variables. The figure shows the importance ranking of feature variables filtered by Boruta in the optimal model (RF). The y-axis displays each feature variable, while the x-axis represents the contribution level of its corresponding outcome variable.

**Figure 6:**
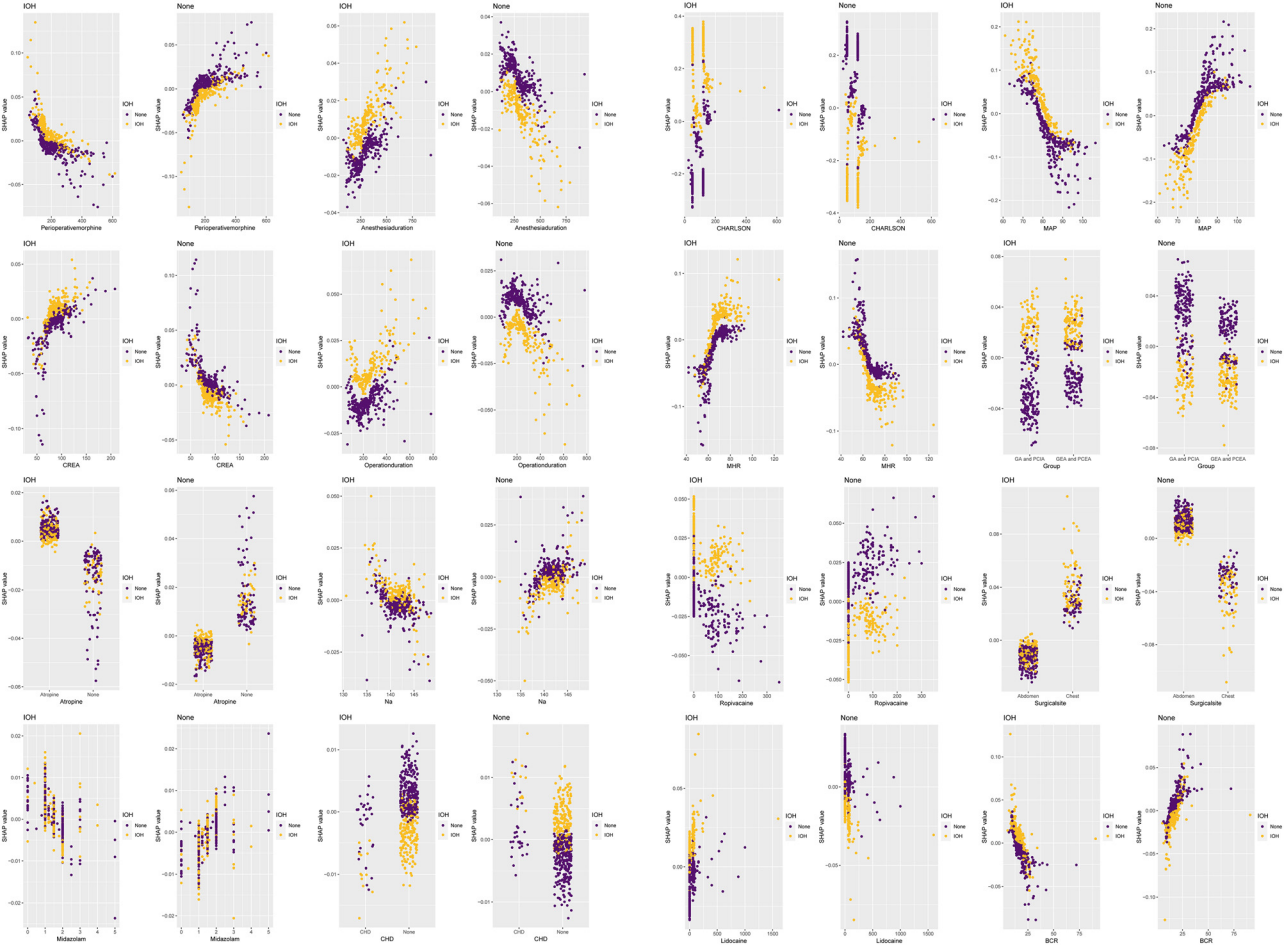
Partial dependency graph of characteristic variables. This graph shows the quantitative trend of each feature variable filtered by Boruta for the outcome variable in the optimal model (RF). The y-axis displays the probability of occurrence of outcome variable, while the x-axis represents the value of its corresponding feature variable.

The random forest (RF) model generated a feature importance ranking plot (Figure 5) and partial dependence plots (Figure 6) for the 16 input variables. The importance ranking visually quantifies each feature’s contribution to intraoperative hypotension (IOH) prediction. Partial dependence plots illustrate the marginal effect of individual features on IOH probability and reveal how predicted IOH risk varies with feature values. These plots were used to analyze the RF model, demonstrating the direction of each feature’s influence (positive or negative) and indicating threshold effects where predictions change substantially when features exceed critical values.

**Figure 7:**
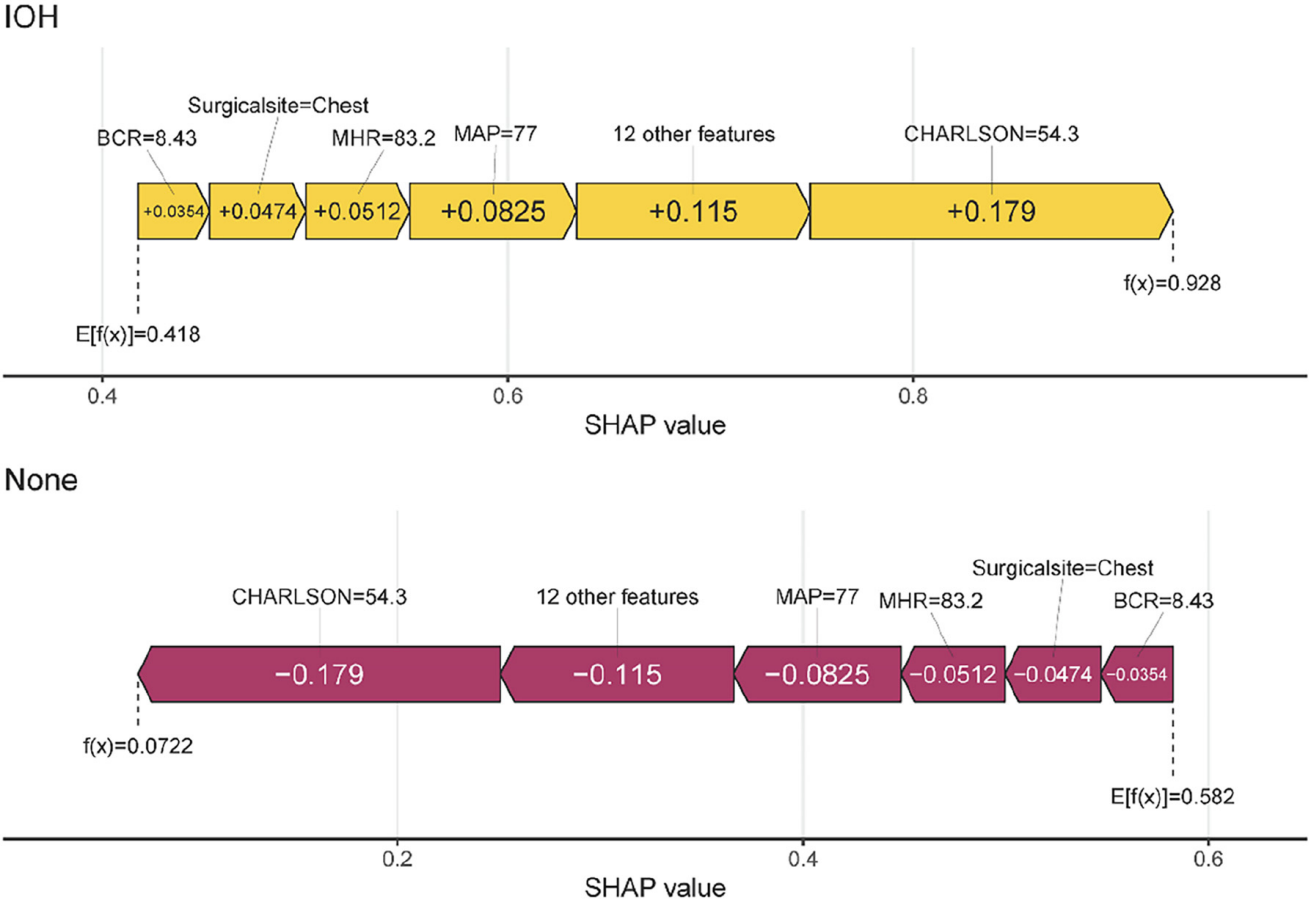
Force plots of the single-sample predictions. Force plots show the visualization process of placing a random sample into the optimal model (RF) for prediction. The graph shows the name, value, and contribution level to the outcome variable of each feature variable in this sample, as well as the model’s the predicted value of the outcome variable.

In this study, the CHARLSON comorbidity index, preoperative MAP (mean arterial pressure), and MHR (mean heart rate) ranked as the top three most significant features. Force plots (Figure 7) visualize individual feature contributions to single-sample predictions. For a sample with actual IOH occurrence (outcome label: IOH), the model predicted an IOH probability of 0.928. Red/yellow bars represent features exerting positive/negative effects on the prediction, with the final predicted value equaling the sum of all feature contributions.

## Discussion

IOH is a common intraoperative complication that can reduce tissue oxygen delivery, leading to organ dysfunction and death [7], 16]. Elderly patients with diminished vascular autoregulation and reduced tolerance to hypotension in vital organs are particularly susceptible to blood pressure fluctuations and subsequent complications [20], 21] such as arrhythmias, myocardial ischemia, pulmonary embolism, renal injury, cerebrovascular damage, cognitive decline, postoperative hemorrhage, and mortality [22], 23]. Identifying high-risk factors in this population is critical to ensure surgical safety and enhance postoperative recovery.

From the feature importance ranking, the Charlson comorbidity index (CCI) emerged as the strongest predictor of IOH [24], followed by preoperative MAP, MHR [25], and anesthesia protocol [26]. The positive association between CCI and IOH may stem from stress responses induced by underlying comorbidities (e.g., hypertension and diabetes) that impair cardiac output, reduce vascular elasticity, or compromise cardiovascular regulation, predisposing patients to hemodynamic instability [27], 28]. Chronic medication use (e.g., β-blockers, ACE inhibitors/angiotensin receptor blockers) may further exacerbate hypotension during anesthesia-induced vasodilation [29], 30]. Preoperative MAP reflects the baseline circulatory status; values <70 mmHg (as shown in partial dependence plots) significantly increased the risk of IOH, potentially indicating chronic hypovolemia (e.g., dehydration) or autonomic dysfunction (e.g., diabetic neuropathy) [31]. Chronic hypertension, which is characterized by arterial stiffness and right-shifted pressure-flow curves, may also increase the risk of hypotension due to anesthetic vasodilation [32].

An elevated preoperative heart rate (>50 bpm) was correlated with a higher IOH risk, possibly reflecting compensatory mechanisms for hypovolemia, pain/anxiety, or early stage heart failure [33]. However, the anesthetic suppression of sympathetic activity may abolish compensatory tachycardia and precipitate abrupt drops in blood pressure [34]. Epidural anesthesia further increases the risk of hypotension via sympathetic blockade, reducing systemic vascular resistance and venous return, particularly in patients with hypovolemia, cardiovascular disease, or advanced age [35]. Excessive doses or widespread use of local anesthetics (e.g., lidocaine and ropivacaine) can intensify this effect [36].

Thoracic surgery poses a higher risk of IOH than abdominal surgery, likely due to positional changes (e.g., lateral decubitus) impairing venous return or direct compression of the heart and great vessels [37], 38]. An elevated preoperative BUN/CREA ratio (>20) indicated hypovolemia or renal dysfunction and served as a warning marker for IOH [39]. Such patients require meticulous preoperative volume assessment and intraoperative hemodynamic monitoring.

This study innovatively combines univariate analysis with the Boruta algorithm for feature selection [40]. Boruta generates “shadow attributes” for each variable, iteratively comparing their Z-scores via random forest to retain only statistically significant predictors. The mlr3 package (a next-generation R toolkit) was used for streamlined data preprocessing, model training (including supervised learning for imbalanced data), repeated cross-validation, hyperparameter tuning, and SHAP-based interpretability. To address class imbalance, random oversampling was used to synthesize minority-class samples, whereas repeated k-fold cross-validation (k=5, n=10 repeats) was used to ensure robust performance estimation [41].

The RF model was used to handle imbalanced data by aggregating multiple weak classifiers into a strong ensemble and inherently prioritizing minority-class samples. External validation was performed to avoid overreliance on accuracy (misleading for imbalanced data), and confusion matrix metrics (AUC-ROC, AUC-PRC, and Matthews correlation coefficient) were calculated for a comprehensive evaluation [42].

SHAP was used to illuminate the “black box” of RF predictions. Feature importance plots ranked predictors were ranked by contribution using feature importance plots, partial dependence plots were used to illustrate the marginal effects of individual features on IOH risk, and force plots were used to represent the influence of specific features on predictions for individual cases (92.8 % predicted IOH probability). This interpretability enhances clinical trust and guides targeted interventions.

The following limitations of this study may have affected our results. First, this study was developed and validated using data from a single center. Although internal validation employed a prospective time-split strategy to assess the model’s temporal stability, the lack of an external, multicenter, geographically diverse validation cohort limits the model’s generalizability across different healthcare systems and patient populations. This represents a critical step that must be addressed before the model can be translated into practical application. Second, we acknowledge that even large, high-quality databases are subject to the inherent limitations of retrospective data, such as missing key variables, measurement variability due to changes in equipment or recording standards over time, and the potential impact of unmeasured confounders on model performance. While we have employed methods like imputation and sensitivity analysis to handle missing data wherever possible, this fundamental limitation still warrants cautious interpretation. Finally, we specifically note that our study cohort excluded emergency surgeries and specific types of cardiac surgeries. Therefore, it remains unknown whether and how the predictive performance of the model can be generalized to these high-risk populations with more complex physiological states and anesthesia management, which points to a clear direction for future targeted research.

## Conclusions

This study developed and validated a machine learning model to predict IOH in 1,720 elderly patients undergoing non-cardiac thoracic/abdominal surgery. By analyzing 94,600 data points, we identified critical predictors (e.g., CCI, MAP, and surgical site) and proposed a clinically actionable framework. Future studies should focus on multicenter validation and real-time integration of perioperative monitoring systems.
